# Regional spontaneous brain activity abnormalities in ischemic stroke patients with motor dysfunction: protocol for an AES-SDM coordinate-based meta-analysis

**DOI:** 10.3389/fneur.2026.1933104

**Published:** 2026-10-09

**Authors:** Jia Zhao, Yao Rao, Jiali Wu, Ming Zeng, Jingjing Zhang, Jiawei Ni, Hui Xu, Zhiwei Zhao, Yaxin Zheng, Cong Wang, Chunlei Shan

**Affiliations:** 1 Department of Rehabilitation Medicine, Tongren Hospital, Shanghai Jiao Tong University School of Medicine, Shanghai, China; 2 Yuanshen Rehabilitation Institute, Shanghai Jiao Tong University School of Medicine, Shanghai, China; 3 National Research Centre for Language and Well-being, Shanghai Jiao Tong University, Shanghai, China

**Keywords:** AES-SDM, coordinate-based meta-analysis, ischemic stroke, motor dysfunction, regional spontaneous brain activity, resting-state fMRI

## Abstract

**Introduction:**

Most ischemic stroke survivors experience motor deficits, yet the underlying patterns of spontaneous brain activity change remain unclear. Individual rs-fMRI studies on this population have yielded contradictory findings, likely due to small sample sizes and between-study differences in lesion profiles, stroke stage, rehabilitation history, and imaging pipelines. These inconsistencies have not been resolved by existing reviews, many of which combine ischemic and hemorrhagic stroke, pool conceptually distinct rs-fMRI metrics without sufficient stratification, or focus on functional connectivity rather than local spontaneous activity. This protocol plans a coordinate-based meta-analysis restricted to ischemic stroke and local rs-fMRI metrics to address that gap.

**Methods and analysis:**

This study will systematically identify and synthesise whole-brain rs-fMRI studies that compared ALFF, fALFF, or ReHo between adult ischemic stroke patients with motor dysfunction and healthy controls. We will search PubMed, Embase, Web of Science, and the Cochrane Library up to 6 February 2026. To be included, a study must report whole-brain voxel-wise group comparisons and provide stereotactic peak coordinates in MNI or Talairach space. Studies will be excluded if they are non-original reports, case reports or case series, use task-state fMRI or ROI-only analyses, lack extractable standard-space coordinates, involve duplicate or overlapping datasets, include hemorrhagic or mixed stroke without separable ischemic-stroke data, or do not provide clearly extractable motor dysfunction-related data. Two reviewers will independently screen, extract, and assess methodological quality. Using anisotropic effect-size seed-based d mapping (AES-SDM), the quantitative synthesis will pool coordinates to detect convergent patterns of both increased and decreased regional spontaneous activity. Where data allow, we will conduct subgroup analyses, jackknife sensitivity testing, heterogeneity assessment, and meta-regression. The protocol follows the PRISMA-P statement, and the completed review will adhere to PRISMA 2020.

**Conclusion:**

This protocol outlines a systematic review and AES-SDM coordinate-based meta-analysis aimed at synthesizing regional spontaneous activity abnormalities in ischemic stroke patients with motor dysfunction. The results should identify convergent areas of local functional alteration, generating testable predictions for future research on post-stroke motor-network reorganization and rehabilitation.

**Clinical trial registration:**

https://www.crd.york.ac.uk/PROSPERO/view/CRD420261359426, identifier CRD420261359426.

## 1 Introduction

Stroke remains a major cause of mortality and long-term neurological disability globally (1). Ischemic stroke accounts for most cases, and many survivors live with persistent functional deficits despite advances in acute reperfusion and organised stroke care (2, 3). Motor dysfunction is among the most common and consequential of these deficits (4, 5). Depending on lesion location, volume, and time since onset, patients may present with hemiparesis, loss of dexterity, abnormal synergies, gait disturbance, impaired balance, or reduced postural control (5, 6). These impairments limit independence, community participation, and return to work, underscoring the need for a clearer understanding of the neural mechanisms that drive post-stroke motor recovery (7, 8).

Functional recovery after ischemic stroke is closely related to neuroplastic changes within distributed motor-related systems, including the primary motor cortex, supplementary motor area, premotor cortex, basal ganglia, thalamus, cerebellum, and associative cortical regions (9, 10). Post-stroke reorganisation can be maladaptive; observed changes may reflect compensatory recruitment, inefficient processing, diaschisis, or maladaptive interhemispheric imbalance (11, 12). Distinguishing these possibilities is essential if neuroimaging findings are to guide prognostic assessment or help identify targets for individualized rehabilitation and non-invasive brain stimulation. Resting-state functional magnetic resonance imaging (rs-fMRI) provides a practical approach for investigating intrinsic brain activity without explicit task demands, which is particularly relevant for stroke patients whose task performance may be limited by weakness, fatigue, aphasia, cognitive impairment, or poor compliance (13, 14). Among rs-fMRI indices, amplitude of low-frequency fluctuations (ALFF) reflects the amplitude of spontaneous low-frequency blood oxygen level-dependent oscillations (15), fractional amplitude of low-frequency fluctuations (fALFF) quantifies the relative contribution of low-frequency fluctuations and is generally considered less susceptible to nonspecific physiological noise, and regional homogeneity (ReHo) indexes the local synchrony of neighboring voxels. Together, these measures provide complementary information about regional spontaneous activity and local functional coherence, and they have been widely applied in studies of post-stroke brain dysfunction and recovery (16). However, these measures capture different properties of local resting-state activity: ALFF/fALFF primarily reflect the amplitude of spontaneous BOLD fluctuations, whereas ReHo reflects local temporal synchronisation (7, 8). Pooling them therefore does not assume a single underlying physiological abnormality; rather, it assesses convergence of anatomical location rather than convergence of the same physiological abnormality. This approach has been adopted in recent coordinate-based meta-analyses, including a meta-analysis of resting-state fMRI studies in ischemic stroke (17).

Despite their increasing use, findings from individual rs-fMRI studies of ischemic stroke patients with motor dysfunction remain inconsistent. Some studies have reported decreased spontaneous activity within ipsilesional sensorimotor regions, whereas others have emphasized increased activity in contralesional homologous areas or in secondary motor and cerebellar regions (18). Similar inconsistency is seen in ReHo findings, with both increases and decreases reported across overlapping anatomical territories (19). These discrepancies are not surprising. Most individual neuroimaging studies remain modest in sample size, and their findings can be influenced by differences in stroke chronicity, lesion laterality, lesion burden, rehabilitation exposure, medication status, scanner parameters, preprocessing pipelines, statistical thresholds, and the specific rs-fMRI metric selected for analysis.

Although previous reviews have summarised stroke-related functional imaging abnormalities, some have combined ischemic and hemorrhagic stroke, pooled distinct rs-fMRI measures without sufficient stratification, or focused mainly on interregional functional connectivity or task-evoked activation, rather than local intrinsic spontaneous activity (14, 20, 21). The key distinction is therefore not the spatial scale of analysis (voxel-wise vs. network-level), but the type of functional measure being investigated. A focused synthesis of convergent voxel-wise abnormalities in local spontaneous activity among ischemic stroke patients with motor dysfunction is therefore still needed. A coordinate-based meta-analysis using anisotropic effect-size seed-based d mapping is well suited to address this gap (22, 23). Compared with approaches that consider only the spatial convergence of reported peaks, Anisotropic effect-size seed-based d mapping (AES-SDM) preserves both the direction and magnitude of reported effects, reconstructs voxel-wise effect-size maps, and enables formal assessment of heterogeneity, sensitivity analyses, and meta-regression of potential moderators. These features are particularly relevant in stroke neuroimaging, where both increased and decreased regional measures may carry biological or clinical significance (17, 22). A dedicated synthesis restricted to ischemic stroke and to local rs-fMRI metrics should therefore yield a more coherent account of regional spontaneous brain activity abnormalities than broader, more heterogeneous reviews.

Against this background, the present protocol aims to systematically evaluate the available rs-fMRI evidence and quantitatively identify brain regions showing convergent abnormalities in regional spontaneous activity and local functional coherence in ischemic stroke patients with motor dysfunction. By focusing on a more clinically defined population and pre-specifying a rigorous coordinate-based analytic strategy, this planned review may provide a more interpretable evidence base for understanding post-stroke motor-network reorganization. In addition, the findings may help generate hypotheses regarding candidate neuroimaging markers for motor recovery, prognosis, and future neuromodulation or rehabilitation-targeting studies.

### 1.1 Objectives

To identify, using AES-SDM, convergent voxel-wise patterns of abnormal regional spontaneous brain activity measured by ALFF, fALFF, and ReHo in adult ischemic stroke patients with motor dysfunction compared with demographically matched healthy controls.

1. To systematically summarize study design features, participant baseline characteristics, key clinical variables, rs-fMRI acquisition parameters, data preprocessing strategies, and primary findings of all included studies.
2. To examine potential sources of between-study heterogeneity, including stroke stage, lesion laterality, motor impairment severity, and rs-fMRI metric, through prespecified subgroup analyses and voxel-wise meta-regression where sufficient data are available.
3. To assess the robustness and reproducibility of the primary findings through jackknife sensitivity analysis and related sensitivity analyses.
4. To evaluate the methodological quality and reporting transparency of the included studies using established tools for observational research and neuroimaging reporting quality.
5. To generate a clinically relevant synthesis that may refine current understanding of regional aspects of post-stroke motor-related brain reorganization and help identify candidate neuroimaging markers for future rehabilitation and neuromodulation research.

## 2 Methods and analysis

The meta-analysis protocol has been registered with the international PROSPERO under the registration number CRD420261359426, and was developed in accordance with Preferred Reporting Items for Systematic review and Meta-Analysis Protocols (PRISMA-P) (24) (Supplementary Data S1).

### 2.1 Eligibility criteria

The pre-specified inclusion and exclusion criteria for study selection are defined as follows.

### 2.2 Study types

This systematic review and coordinate-based meta-analysis will exclusively include nonrandomized study designs, specifically original human neuroimaging research articles employing a case–control or cross-sectional observational design. To be included, a study must provide whole-brain voxel-wise resting-state fMRI analyses that yield significant peak coordinates in standard stereotactic space (Montreal Neurological Institute [MNI] or Talairach), with extractable data for at least one of the core local activity metrics: ReHo, ALFF, or fALFF. Only peer-reviewed full-text articles will be considered. Studies will be excluded if they meet any of the following criteria: (1) reviews, systematic reviews, meta-analyses, editorials, letters, or conference abstracts without full text; (2) case reports, single-case studies, case series, or studies with fewer than five participants per group; (3) task-state fMRI studies, ROI-only studies lacking whole-brain data, or studies without extractable standard-space peak coordinates for the core metrics; (4) duplicate publications or studies with overlapping participant samples, in which case only the publication reporting the largest or most complete dataset will be retained; and (5) pediatric studies, animal studies, or *in vitro* studies.

### 2.3 Participants

Eligible participants will include adults aged 18 years or older with a clinical diagnosis of ischemic stroke confirmed by structural neuroimaging (computed tomography [CT] or magnetic resonance imaging [MRI]) and clearly defined motor dysfunction verified by clinical description or standardized motor assessment scales, including but not limited to the Fugl-Meyer Assessment, motor items of the National Institutes of Health Stroke Scale (NIHSS), Brunnstrom stages, Motricity Index, Medical Research Council scale, or other validated motor assessments. We will not exclude patients based on sex, race, handedness, lesion location, or time since stroke (acute, subacute, or chronic).

Exclusion criteria include hemorrhagic stroke, mixed stroke, traumatic brain injury, brain tumors, neurodegenerative diseases, and other non-ischemic etiologies. Studies will also be excluded if ischemic stroke-specific data cannot be separated from mixed samples. Additional exclusion criteria include studies primarily focused on aphasia, cognitive impairment, depression, or other non-motor outcomes without clear and extractable motor dysfunction–related data, as well as pediatric studies, animal studies, and *in vitro* studies.

### 2.4 Exposure

The exposure of interest in this review will be rs-fMRI-derived local activity measures in patients with ischemic stroke-related motor dysfunction. Included studies must evaluate at least one of the three core metrics of local spontaneous neural activity: ReHo, ALFF, or fALFF. Eligible studies are required to report whole-brain voxel-wise analysis results with extractable peak coordinates in a standard stereotactic space (MNI or Talairach).

Studies involving task-state fMRI, those exclusively based on ROI without whole-brain data, and those lacking extractable coordinates for the core metrics (ReHo, ALFF, or fALFF) will be excluded. For studies reporting both ROI and whole-brain results, only the whole-brain data will be extracted for meta-analysis. These criteria are intended to reduce methodological heterogeneity and improve the interpretability of convergent regional brain functional abnormalities in patients with ischemic stroke-related motor dysfunction. Studies reporting additional neuroimaging measures will be eligible only when they also provide whole-brain ALFF, fALFF, or ReHo results with extractable stereotactic coordinates.

### 2.5 Comparators

Eligible comparators are healthy adults aged ≥18 years with no reported history of neurological or psychiatric disorders and no known motor or major cognitive impairment. The control group should be demographically comparable to the ischemic stroke patient group, particularly with respect to age and sex where reported. Studies in which the control group is clearly not matched or includes participants with the above conditions will be excluded from the primary quantitative synthesis. If the adequacy of demographic matching or comparability cannot be determined because of incomplete reporting, this information will be recorded during methodological quality assessment and considered in sensitivity analyses where data permit.

### 2.6 Outcome measures

The primary outcome will be the whole-brain spatial convergence pattern of abnormal regional spontaneous brain activity in ischemic stroke patients with motor dysfunction compared with healthy controls, as measured by three core resting-state fMRI local activity metrics: amplitude of low-frequency fluctuations, fractional amplitude of low-frequency fluctuations, and regional homogeneity. Significant peak coordinates in Montreal Neurological Institute or Talairach space, together with effect directions and statistical values, will be extracted. The primary analysis will pool ALFF, fALFF, and ReHo to identify convergent abnormalities, as has been done in previous meta-analyses (17); metric-specific meta-analytic maps will be generated as preplanned subgroup analyses when a sufficient number of datasets is available.

Secondary outcomes will include study-level associations between regional spontaneous activity alterations and motor function measures, such as the Fugl-Meyer Assessment or other validated motor-related scales, where sufficient data are available. These associations will be explored using voxel-wise meta-regression only when an adequate number of independent datasets provide the relevant clinical variables.

Methodological quality, risk of bias, between-study heterogeneity, publication bias, and robustness of findings will be assessed as part of the planned methodological and statistical analyses rather than as outcome measures.

### 2.7 Search strategy

Two independent reviewers will conduct a comprehensive systematic search across four major electronic databases: PubMed, Embase, Web of Science, and the Cochrane Library, from their inception up to 6 February 2026. Only records indexed in these selected databases will be considered. The search strategy will employ a combination of Medical Subject Headings (MeSH) and relevant free-text keywords, combined using Boolean operators (AND/OR). To improve sensitivity and specificity, the strategy will explicitly pair ischemic stroke-related terms with motor dysfunction-related terms (e.g., “ischemic stroke,” “cerebral infarction,” “hemiplegia,” “hemiparesis,” “motor impairment”), restrict imaging terms to resting-state functional MRI terminology (e.g., “resting-state fMRI,” “rs-fMRI,” “functional magnetic resonance imaging”, “BOLD”), and include specific keywords for the three core local activity metrics (e.g., “regional homogeneity” OR “ReHo”, “amplitude of low-frequency fluctuation” OR “ALFF”, “fractional ALFF” OR “fALFF”). The PubMed search string tailored to this strategy is provided in Supplementary Data S2, and will be adapted for the other databases according to their respective syntax.

## 3 Data collection and analysis

### 3.1 Selection process

Reference management and deduplication will be performed using EndNote X9 software (Thomson Reuters, New York, United States). Following the removal of duplicates, two independent reviewers will screen the titles and abstracts to exclude clearly irrelevant records. Potentially eligible studies will then undergo a comprehensive full-text review to determine final inclusion strictly based on the pre-defined eligibility criteria. Any discrepancies arising during the screening process will first be resolved through discussion between the two primary reviewers; if consensus cannot be reached, a third independent senior reviewer with extensive experience in neuroimaging meta-analysis will be consulted for final adjudication. The entire selection process will be rigorously documented and presented in a PRISMA 2020-compliant flow diagram (Figure 1), which will separately report the number of records identified, screened, assessed for eligibility, and included in the qualitative narrative synthesis and quantitative coordinate-based meta-analysis, to ensure full methodological transparency and reproducibility.

**Figure 1:**
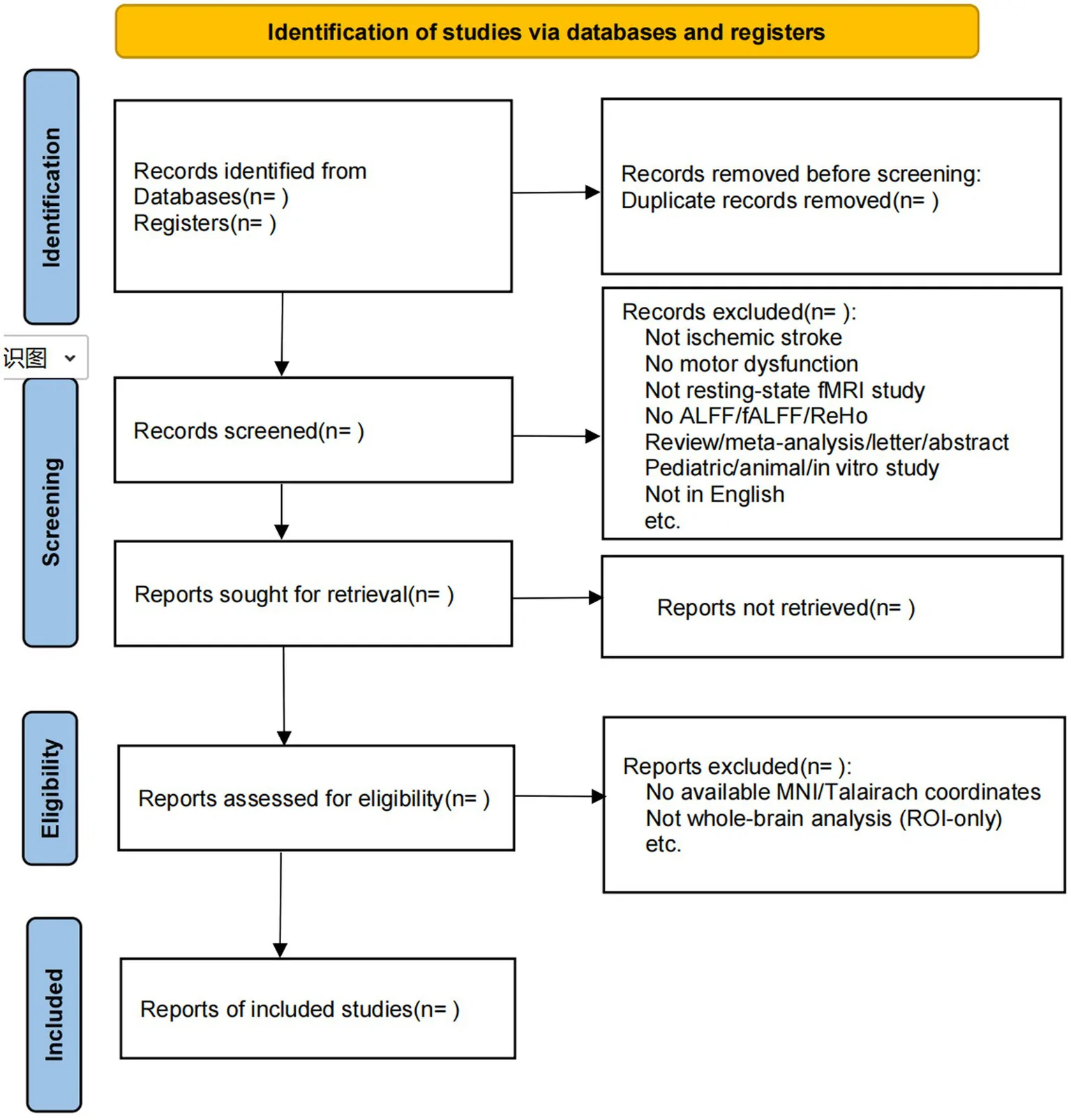
PRISMA 2020 flow diagram for study selection.

### 3.2 Data collection

Two reviewers will independently extract data using a piloted form, which will first be trialled on 10% of eligible studies and refined to improve inter-rater consistency. Disagreements will be resolved by discussion, with arbitration by a third senior reviewer where needed. The form records each eligible contrast’s statistical threshold (corrected or uncorrected), coordinate space, effect direction, and whether results came from whole-brain voxel-wise comparisons, so that conflicting findings can be handled transparently. To avoid unit-of-analysis errors, multiple non-independent contrasts from the same dataset will not be pooled in the primary synthesis:

1. *Study characteristics*: bibliographic information (first author, year of publication, country), study design (case–control or cross-sectional), and sample size (patients and controls).
2. *Participant profile*: mean age, sex distribution, handedness, time post-stroke, lesion characteristics (location and hemisphere), stroke severity, and standardized motor function assessment scores (e.g., Fugl-Meyer Assessment, Brunnstrom stage, motor items of the NIHSS, and Barthel Index).
3. *Imaging acquisition and preprocessing*: scanner field strength, repetition time (TR) and echo time (TE), number of volumes, slice thickness and gap, and the software/pipeline used for image preprocessing and statistical analysis.
4. *Core metric details*: which rs-fMRI indicator was analysed (ALFF, fALFF, or ReHo), the statistical threshold and multiple-comparison correction (e.g., FWE, FDR, or uncorrected), and whether whole-brain voxel-wise analysis was confirmed.
5. *Coordinate outcomes*: stereotactic peak coordinates (x, y, z) of statistically significant clusters for the pre-specified case–control contrasts, coordinate space (MNI or Talairach), direction of effect (patients > controls or patients < controls), and associated statistic values (*t*-value, *z*-score, or *p*-value). Talairach coordinates will be converted to MNI space using the validated Lancaster transformation.

When multiple eligible results are reported from the same study, all relevant results will be recorded, but only one non-overlapping contrast per independent dataset will be included in each primary analysis. For studies reporting multiple eligible metrics, ALFF, fALFF, and ReHo results will be entered into their corresponding metric-specific analyses. For longitudinal or intervention studies, only baseline patient-versus-control data will be extracted. Lesion characteristics, including lesion location, lesion hemisphere, and whether images or coordinates were flipped in the original study, will also be extracted. Coordinates will be analyzed as reported in the original studies. If sufficient data are available, lesion-normalized analyses may be performed as sensitivity analyses. Extracted data will be collated into a “Characteristics of Included Studies” table. Where required data are missing and cannot be obtained from authors, the study will be excluded from the primary quantitative synthesis but may still contribute to the narrative synthesis.

### 3.3 Data synthesis

Extracted data will first be organised into a summary table that records, for each study, the bibliographic information, design, sample and clinical characteristics, motor assessment details, imaging and preprocessing parameters, and rs-fMRI metrics analysed. Although ALFF/fALFF and ReHo reflect different properties of local resting-state activity—ALFF/fALFF primarily reflecting the amplitude of spontaneous BOLD fluctuations and ReHo reflecting local temporal synchronisation—pooling them does not assume a single underlying physiological abnormality. Rather, it assesses convergence of anatomical location across complementary aspects of local activity. When sufficient datasets are available, we will perform metric-specific subgroup analyses for ALFF, fALFF, and ReHo separately and sensitivity analyses to test whether the pooled results are driven by any single metric.

The primary synthesis will be a random-effects coordinate-based meta-analysis (CBMA) using anisotropic effect-size seed-based d mapping (AES-SDM; SDM-PSI, version 5.15 or later). We chose AES-SDM over other CBMA methods because stroke-related activity changes can be bidirectional, with some regions showing increased and others decreased spontaneous activity, and AES-SDM retains the direction and magnitude of each peak. The method also reconstructs voxel-wise effect-size maps from peak coordinates and associated statistics and supports voxel-wise heterogeneity testing and meta-regression. Peak coordinates, effect directions, and associated statistics will be converted to MNI152 space where needed and used to reconstruct whole-brain effect-size maps (Hedges’ *g*) for each study with an anisotropic Gaussian kernel (FWHM = 20 mm). These study-level maps will then be pooled under a random-effects model, with each study weighted by the inverse of its within-study variance plus between-study heterogeneity, following the standard AES-SDM implementation.

Separate statistical maps will be generated for regions where patients showed higher or lower ALFF, fALFF, or ReHo than controls. When enough datasets are available, we will run metric-specific analyses for ALFF, fALFF, and ReHo separately. All AES-SDM analyses will follow current SDM-PSI recommendations. For comparability with prior CBMA studies, we will apply the conventional AES-SDM threshold: voxel-level *p* < 0.005, peak SDM-Z > 1, and cluster extent ≥10 voxels. Sensitivity analyses will use stricter thresholds (e.g., *p* < 0.001, *k* ≥ 50 voxels) to test the stability of results.

Between-study heterogeneity will be assessed voxel-wise with Q statistics and I^2^ maps, using the AES-SDM implementation. *I*^2^ values of 25, 50, and 75% will be taken to indicate low, moderate, and high heterogeneity. Pre-planned subgroup and meta-regression analyses are contingent on data availability: subgroup analyses require at least three independent datasets per level; voxel-wise meta-regression requires the relevant covariate from ten or more datasets.

Studies that meet inclusion criteria but lack extractable stereotactic coordinates will be set aside for narrative synthesis, following the Synthesis Without Meta-analysis (SWiM) guidelines, with their characteristics and findings presented in separate tables. Studies reporting only other imaging measures will not enter the primary CBMA, unless they also provide eligible whole-brain ALFF, fALFF, or ReHo data with usable coordinates.

### 3.4 Quality assessment

To evaluate the methodological quality of the included neuroimaging studies, we will employ a customized 20-point checklist adapted from previous peer-reviewed meta-analyses (25, 26). The checklist evaluates the completeness and transparency of neuroimaging reporting across three domains: sample characteristics (10 points), imaging acquisition (4 points), and statistical analysis (6 points). It is designed as a reporting-quality tool rather than a formal risk-of-bias instrument. Detailed scoring is shown in Table 1.

**Table 1:** Quality assessment of individual studies: criteria for objective methodological evaluation.

Category	Criteria	Score
Category 1: sample characteristics (10)	Patients were evaluated with specific standardized diagnostic criteria	1
	Important demographic data (age and gender) were reported with mean (or median) and standard deviations (or range)	2
	Healthy comparison subjects were evaluated to exclude neurological or psychiatric disorders, and demographic data (age and sex) were reported	1
	Important clinical variables (e.g., time post-stroke, lesion location and laterality, and motor function scores such as Fugl-Meyer Assessment, NIHSS motor items etc.) were reported with mean (or median) and standard deviations (or range)	4
	Sample size per group ≥ 10	2
Category 2: methodology and reporting (10)	Whole brain analysis was automated with no a-priori regional selection	3
	Magnet strength at least 1.5 T	1
	At least 5 min of resting state acquisition	1
	Whole brain coverage of resting scans	1
	The acquisition and preprocessing techniques were clearly described so that they could be reproduced	1
	Coordinates reported in a standard space	1
	Significant results are reported after correction for multiple testing using a standard statistical procedure (FDR, FWE or permutation-based methods)	1
	Conclusions were consistent with the results obtained and the limitations were discussed	1

FMA: Fugl-Meyer Assessment; NIHSS: National institutes of health stroke scale; FDR: false discovery rate; FWE: family-wise error.

Risk of bias will be appraised with the Newcastle-Ottawa Scale (NOS) for case–control studies, alongside a neuroimaging-specific reporting checklist. The NOS will be used to evaluate study selection, comparability of cases and controls, and ascertainment of imaging outcomes, whereas the customized neuroimaging checklist will focus on the completeness and transparency of reporting for sample characteristics, image acquisition, preprocessing, and statistical analysis. The NOS evaluates study quality across three domains: selection of study groups, comparability of cases and controls on key demographic and clinical confounders, and ascertainment of the outcome of interest (spontaneous brain activity alterations). Studies scoring ≥ 7 stars will be classified as high quality, 4–6 stars as moderate quality, and < 4 stars as low quality. Quality and risk of bias assessment will be performed independently by two reviewers, with any disagreements resolved through discussion or consultation with a third independent senior reviewer. Additional information will be sought from study investigators if required details are unclear or unavailable in the published reports. The final quality ratings will be used to inform pre-specified sensitivity analyses (exclusion of low-quality studies) and to contextualize the interpretation of the final meta-analytic findings.

### 3.5 Sensitivity analysis

The robustness of the primary CBMA results will be tested with a leave-one-out jackknife analysis. In this procedure, the CBMA will be repeated iteratively, with one independent dataset omitted in each iteration. A brain region will be considered methodologically robust if it remains statistically significant across ≥80% of all jackknife iterations. Additional pre-specified sensitivity analyses will include: (1) application of more conservative statistical thresholds (e.g., voxel-level *p* < 0.001, cluster extent ≥ 50 voxels); (2) exclusion of studies rated as low quality (NOS score < 4 stars); and (3) excluding studies with unclear sample independence or potentially overlapping patient samples, where applicable. Sensitivity analyses will be undertaken only when the number and design of available datasets make such testing statistically meaningful; therefore, not every planned sensitivity procedure will necessarily be applicable to every outcome or subgroup.

### 3.6 Heterogeneity analysis

Between-study heterogeneity will be assessed using the Q statistic and *I*^2^ statistic, with all analyses performed within the pre-specified random-effects meta-analytic framework. Voxel-wise heterogeneity maps will be calculated within AES-SDM to identify brain regions with significant variability in effect sizes across included studies. I^2^ values of 25, 50, and 75% will be interpreted as indicating low, moderate, and high between-study heterogeneity, respectively. Statistically significant heterogeneity (defined as *p* < 0.05 for the Q statistic) will warrant cautious interpretation of the corresponding brain clusters. Potential sources of significant heterogeneity will be further explored through pre-planned subgroup analyses and voxel-wise meta-regression, as described in Sections 3.7 and 3.8.

### 3.7 Subgroup analysis

Significant between-study heterogeneity (defined as *p* < 0.05 for the Q statistic) warrants cautious interpretation of the identified brain clusters. To explore potential sources of variability in effect sizes within heterogeneous brain regions, we will perform pre-planned subgroup analyses stratified according to: (1) motor impairment severity, classified as mild versus moderate-to-severe based on reported standardised motor impairment scales, using predefined clinical cut-offs where available or study-level median splits where necessary; (2) stroke stage (acute/subacute ≤ 6 months post-stroke versus chronic > 6 months post-stroke); (3) lesion hemisphere (left versus right hemisphere); (4) individual core imaging metric (ReHo versus ALFF versus fALFF); and (5) type of statistical threshold applied (family-wise error/FDR corrected versus uncorrected). Subgroup analyses will be conducted only when a sufficient and reasonably balanced number of independent datasets is available across subgroup levels. As a pragmatic minimum, at least three independent datasets per subgroup level will be required. All subgroup findings will be interpreted cautiously as exploratory and hypothesis-generating.

### 3.8 Meta-regression analysis

To further investigate the association between regional spontaneous brain activity alterations and clinical characteristics, voxel-wise meta-regression analyses will be conducted using the AES-SDM software. Specifically, we will examine the linear relationship between study-level effect sizes of altered brain activity and study-level mean values of continuous clinical variables, including: (1) mean total motor function scores (e.g., Fugl-Meyer Assessment scores); (2) mean age of included participants; and (3) mean time post-stroke. Meta-regression analyses will be performed only when data for the relevant covariates are available from at least 10 independent studies, to ensure sufficient statistical power and reduce the risk of spurious findings due to overfitting. Because meta-regression will be based on study-level averages, findings will be interpreted as ecological associations rather than individual-level relationships.

### 3.9 Narrative synthesis

Studies that meet the clinical eligibility criteria but are unsuitable for the primary quantitative CBMA will be summarized narratively. These may include studies without extractable standard-space peak coordinates for the core metrics; such studies will be excluded from the primary quantitative CBMA but may still be considered for narrative synthesis if they otherwise meet the clinical and imaging eligibility criteria. Narrative synthesis will be conducted in accordance with the SWiM reporting guidance where appropriate. The synthesis will focus on the direction and anatomical distribution of reported ALFF, fALFF, or ReHo alterations, together with relevant study characteristics, including sample size, participant profile, imaging acquisition parameters, preprocessing strategies, and statistical thresholds. Other neuroimaging measures will not be treated as predefined outcomes of this review.

### 3.10 Publication bias

Publication bias will be assessed for brain clusters exhibiting statistically significant alterations in the primary CBMA. This will be performed via visual inspection of funnel plots and quantitative Egger’s regression test, as implemented in the AES-SDM software. The analysis will be conducted specifically for the peak coordinates of the identified significant clusters. As these tests have limited statistical reliability with sparse datasets, they will only be applied when a sufficient number of independent datasets contribute to the analysis (preferably ≥ 10 independent datasets). A *p*-value < 0.05 in Egger’s test will be considered indicative of statistically significant publication bias or small-study effects. In cases where the number of contributing datasets is insufficient for formal statistical testing, we will transparently report this limitation and discuss its potential impact on the interpretation of findings.

### 3.11 Confidence in cumulative evidence

A formal GRADE (Grading of Recommendations Assessment, Development and Evaluation) assessment will not be applied in this study, as coordinate-based neuroimaging biomarker outcomes do not directly map onto the GRADE framework’s core focus on patient-important clinical intervention effect estimates. Instead, confidence in the cumulative evidence will be systematically inferred from four key domains: (1) the overall methodological quality of included studies, as assessed by the NOS and the customized 20-point neuroimaging reporting checklist; (2) the consistency of convergent abnormal brain regions across independent included studies; (3) the magnitude and statistical significance of observed between-study heterogeneity; and (4) the robustness of the primary findings across all pre-specified sensitivity analyses (jackknife leave-one-out analysis, alternative conservative thresholds, and exclusion of low-quality studies).

### 3.12 Patient and public involvement

Patients and the public will not be involved in the design, conduct, reporting, or dissemination plans of this systematic review and meta-analysis protocol, or in the execution of the planned study.

### 3.13 Ethics and dissemination

Formal ethical approval is not required for this study, as it is a systematic review and coordinate-based meta-analysis that synthesizes data exclusively from previously published, peer-reviewed literature. No new individual participant data will be collected, no primary human subjects research will be conducted, and there will be no direct intervention or interaction with human subjects. The final results of this systematic review and meta-analysis will be submitted for publication in a peer-reviewed international journal and disseminated through academic conferences.

## 4 Discussion

This protocol describes a systematic review and coordinate-based meta-analysis (CBMA) designed to identify consistent patterns of regional spontaneous brain activity alterations in ischemic stroke patients with motor dysfunction, using three canonical rs-fMRI metrics: ReHo, ALFF, and fALFF. The protocol has been developed in accordance with the PRISMA-P 2015 statement (24) and addresses several important limitations in the existing literature.

A recent coordinate-based meta-analysis of general ischemic stroke identified convergent abnormalities in ALFF, fALFF, and ReHo (17), but did not restrict inclusion to patients with motor dysfunction. Another meta-analysis examined functional connectivity in post-stroke motor dysfunction but addressed inter-regional connectivity rather than local spontaneous activity (27). Existing reviews have also frequently combined ischemic and hemorrhagic stroke without stratified analysis (21), despite well-documented differences in pathophysiology, motor recovery trajectories, and brain network reorganization between the two subtypes (11, 16). Even among reviews focusing on post-stroke motor outcomes (20), a quantitative synthesis specifically restricted to regional spontaneous activity metrics in ischemic stroke remains absent. Furthermore, most existing CBMA studies in this field have used activation likelihood estimation (ALE) methods (28), which cannot incorporate the direction and magnitude of effects within a single model. The planned review will therefore provide a dedicated AES-SDM synthesis of ALFF, fALFF, and ReHo abnormalities in ischemic stroke patients with motor dysfunction, while explicitly accounting for metric-specific differences and sources of clinical and methodological heterogeneity.

Several design features help address these limitations. First, by restricting inclusion to ischemic stroke patients with motor dysfunction, we reduce the clinical heterogeneity that complicates mixed-stroke reviews. The results should therefore be more directly relevant to the majority of stroke survivors living with motor disability. Second, the pre-specified separate analysis for each of the three core rs-fMRI metrics maximizes construct homogeneity, as ReHo, ALFF, and fALFF reflect distinct aspects of local brain activity (7, 8). When these measures are pooled in the primary analysis, convergence should be interpreted as convergence of anatomical location rather than convergence of the same physiological abnormality. If sufficient studies are available, measure-specific subgroup and sensitivity analyses will be conducted to strengthen this interpretation. Third, the use of AES-SDM provides significant methodological advantages over traditional ALE approaches: it preserves both the magnitude and direction of effect sizes, enables voxel-wise heterogeneity assessment and meta-regression, and allows estimation of effect sizes from non-significant peaks to reduce the impact of selective reporting (29, 30). Finally, the pre-registration of this protocol in PROSPERO, along with the pre-specified analysis plan, enhances transparency and reproducibility of the research process. However, AES-SDM may underestimate convergence when a region shows opposite effect directions across studies. We will visually inspect clusters for such patterns and discuss them if found.

Several potential limitations of this study should be acknowledged, along with pre-specified strategies to mitigate their impact. First, the number of eligible studies for specific subgroup and meta-regression analyses may be limited, which could restrict statistical power. To address this, we will transparently report the feasibility of each pre-specified analysis, and interpret all exploratory results with appropriate statistical caution, in accordance with standard CBMA methodological recommendations (30, 31). Second, between-study heterogeneity may be introduced by variability in patient characteristics, image acquisition parameters, and preprocessing pipelines. We will systematically explore potential sources of heterogeneity through pre-planned subgroup analyses and meta-regression, and quantify heterogeneity at the voxel level using standard Q and I^2^ statistics. Third, because the search will be restricted to four selected international databases, relevant studies not indexed in these databases may be missed. This limitation will be acknowledged when interpreting the completeness and generalizability of the evidence base. Fourth, studies without usable standard-space stereotactic coordinates will be excluded from the primary quantitative CBMA, which may lead to the loss of some relevant data. To mitigate this, we will include these studies in a systematic narrative synthesis adhering to the SWiM reporting guidelines, to maintain a comprehensive overview of the full evidence base. Fifth, the two recent meta-analyses most closely related to this work (17, 27) were published outside our search window and will not be included in the quantitative synthesis; their findings will instead serve as key comparators in interpreting our results. Finally, as a meta-analysis of observational cross-sectional studies, the findings will identify consistent associations between regional brain activity alterations and motor dysfunction, but cannot establish causal relationships.

The completed review may have implications for both neuroimaging research and stroke rehabilitation. From a scientific perspective, it may help clarify inconsistencies in the existing literature and identify candidate regions involved in post-stroke motor-related brain reorganization. Together with recent work on functional connectivity in this population (27), the review may help clarify how local spontaneous activity changes relate to broader network-level alterations after ischemic stroke. From a rehabilitation perspective, identifying reproducible patterns of altered regional spontaneous brain activity may improve our understanding of the neural mechanisms underlying motor impairment and recovery after ischemic stroke. These convergent abnormalities may provide candidate neuroimaging markers for future studies of patient stratification, recovery prognosis, treatment-response monitoring, and individualized rehabilitation or non-invasive neuromodulation strategies.
